# Temporal structures that determine consistency and quality of care: a case study in hyperacute stroke services

**DOI:** 10.1136/bmjqs-2022-015620

**Published:** 2023-06-19

**Authors:** Georgia B Black, Angus I G Ramsay, Robert Simister, Abigail Baim-Lance, Jeannie Eng, Mariya Melnychuk, Naomi J Fulop

**Affiliations:** 1 Applied Health Research, University College London, London, UK; 2 Wolfson Institute of Population Health, Queen Mary University of London, London, UK; 3 Stroke Research Centre, Institute of Neurology, University College London, London, UK; 4 Geriatrics and Palliative Medicine, Mount Sinai School of Medicine, New York, New York, USA; 5 James J Peters VA Medical Center, US Department of Veterans Affairs, New York, New York, USA

**Keywords:** Quality improvement, Qualitative research, Health services research

## Abstract

**Background:**

*Temporal structuring is determined by practices and social norms and affects the quality and timing of care*. In this case study of hyperacute stroke wards which provide initial stroke investigation, treatment and care, we explored temporal structuring patterns to explain how these may affect quality of care.

**Methods:**

This paper presents a thematic analysis of qualitative interviews with hyperacute stroke staff (n=76), non-participant observations (n=41, ~102 hours) and documentary analysis of the relevant service standards guidance. We used an inductive coding process to generate thematic findings around the concept of temporal structuring, with graphically illustrated examples.

**Results:**

Five temporal structures influence what-happens-when: (1) clinical priorities and quality assurance metrics motivate rapid activity for the initial life-prolonging assessments and interventions; (2) static features of ward organisation such as rotas and ward rounds impact consistency of care, determining timing and quality of care for patients; (3) some services experimented with staff rotas to try to meet peaks in demand, sometimes unsuccessfully; (4) implicit social norms or heuristics about perceived necessity affected staff motivation to make changes or improvements to consistency of care, particularly around weekend work; and (5) after-effects such as bottlenecks or backlogs affect quality of care, which are hard to measure effectively to drive service improvement.

**Conclusions:**

Patients need temporally consistent high quality of care. Temporal consistency stems from the design of services, including staffing, targets and patient pathway design as well as cultural attitudes to working patterns. Improvements to consistency of care will be limited without changes to structures such as rotas and ward rounds, but also social norms around weekend work for certain professional groups.

**WHAT IS ALREADY KNOWN ON THIS TOPIC**

- Previous studies have established that temporal structuring (the what-happens-when) of clinical activities affects quality of care, such as the amount of time nurses are able to spend with patients.
- Variation in stroke care persists due to differences in professional staffing by time of day, delivery of therapy assessments and barriers to discharging patients.

**WHAT THIS STUDY ADDS**

- Temporal consistency stems from the design of services, including staffing, targets and patient pathway design as well as cultural attitudes and norms setting to working patterns.

**HOW THIS STUDY MIGHT AFFECT RESEARCH, PRACTICE OR POLICY**

- Improvements in consistency will require changes in attitudes as well as investment, for example, extending therapy services at weekends requires additional resources and not just changes to rotas.
- Researchers exploring temporal consistency of care quality should attend to how services are organised and not just rely on routinely collected or audit data.

## Introduction

The public need to receive care of the same quality whenever they receive it,^1^ yet it has been recognised internationally that healthcare systems do not function in the same way on each day of the week or during the night compared with daytime hours. Therefore, *temporal consistency* has been established as a marker for quality of care, explored at length in studies relating to 7-day services or the ‘weekend effect’.^2 3^

Researchers have measured quality of care by comparing weekend and night-time (‘out of hours’) against daytime,^4 5^ and weekend against weekday,^6^ as well as looking at patterns of *temporal variation*.^7–10^ The latter approach suggests that certain times of the day, or days of the week, are associated with poorer outcomes for the patient but that this is not limited to the weekend or night-time.^7^ As such, the concept of out-of-hours working, which once dominated the discourse of the literature on temporal variation, has been re-evaluated.^4 7 11^

We have previously published findings about how stroke services produce consistent outcomes despite variation in staffing and resources and the unintended consequences.^12 13^ This revealed that services must deploy their efforts disproportionately on those aspects that have the greatest impact on clinical outcomes, employing strategies such as stretching junior staff roles at night to achieve consistency.^12^ Bion *et al* conducted a similar study in an acute setting, examining emergency admissions across different time periods, and found that temporal consistency is not a sufficient marker for quality, rather consistently *high* performance.^14^

More work is needed to understand variation in the organisation of care over time and to ensure that ways of monitoring consistency of care are sensitive to care quality.^2^ In this paper, we use the term *temporal structuring* to describe regular or changing temporal patterns that affect quality and timing of care, determined both by staff practices and local ideas about what is ‘normal’ work at any given time.^15^ Our aim is to describe the social structures and normative attitudes that influence the timing of clinical processes using the case study of hyperacute stroke units (HASUs).

## Methods

The work presented in this paper was included in a previously published report of a mixed methods study looking at temporal variation in hyperacute stroke care.^16^ The current paper has been abridged and oriented more closely to audiences concerned with drivers of quality of care. This paper draws on a primary qualitative analysis of staff interviews and observations and refers to some quantitative findings reported in previous publications.^12 13^ Our study team includes health service researchers and stroke clinicians.

### Setting

This study was based across eight HASUs in London. These units provide specialised stroke care to all patients with suspected acute stroke in a centralised model, allowing for specialist stroke team assessment, immediate brain imaging and, when appropriate, immediate intravenous thrombolysis.^17^ The HASUs aim to provide specialised care during the first 72 hours after the onset of stroke, after which patients are transferred to 1 of 24 acute stroke units for ongoing care, if required.^17^ Outcomes for patients who had a stroke depend on fast, coordinated treatment due to the loss of nervous tissue over time;^18^ during the critical therapeutic window in the first 24 hours after onset, evidence suggests that multidisciplinary input including medical, nursing, speech and language, clinical psychology, physiotherapy and occupational therapy reduces disability and saves lives.^19^

### Study design

Our full study included Sentinel Stroke National Audit Programme (SSNAP) data, patient interviews, staff interviews, observations and documents. In this paper, we report on thematic analysis of a subset of this dataset, namely,

- Staff interviews (n=76) (see table 1).
- Observations (n=45, ~102 hours) (see table 2).
- Documentary analysis.

**Table 1:** Summary of activities observed during non-participant observations (reproduced with permission from Simister *et al* 2020)^16^

Activities observed	Total (of eight sites)
Patient arrival at the emergency department	7/8
Discussion of individual patient’s care at the ward round	6/8
Multidisciplinary team meetings	7/8
16:00 catch-up meeting on the ward	3/4*
Nurse handovers when staff from one shift leaves and another arrives	8/8
Bed meetings to discuss potential patients to be discharged	6/8
Patients being discharged including paperwork and waiting for transport or family members to arrive	4/8
Times observed	Total
In hours observations	25
Night observations	8
Weekend observations	8
Total observation periods conducted	41

*Four hyperacute stroke units have this activity.

**Table 2:** Staff participants by site and occupation (reproduced with permission from Simister *et al*)^16^

Profession	HASU site identifier								
	H1	H2	H3	H4	H5	H6	H7	H8	Total
Consultant*	2	1	2	1	1	2	1	1	11
Registrar	1	1	1	1		1	1	2	8
Senior nurse/ward manager	1		1	1	1	1	1	1	7
Senior house officer	1	1	1	1	1	1	1		7
Occupational therapist	1	1	1	1	1	1	1	1	8
Physiotherapist	2	1	1	1	2	1	1	1	10
Speech and language therapist	1	1	1	1	1	1	1	1	8
Administrator/SSNAP		1						1	2
Nurse	1	1	1	1	1	1	1	1	8
Stroke coordinator/facilitator	1	1		1	1	1	1		6
HASU lead research nurse								1	1
Total interviews completed	11	9	9	9	9	10	9	10	76

*These numbers include the service lead (consultant) in each site.

HASU, hyperacute stroke unit; SSNAP, Sentinel Stroke National Audit Programme.

Documents included the National Clinical Guideline for Stroke (on which SSNAP is based) and the London Stroke Acute Commissioning and Tariff Guidance.^20 21^ SSNAP guidelines apply to all stroke services in England, Wales and Northern Ireland. London guidance applies only to HASUs in London.^21^

Three researchers (GBB, JE and AB-L) conducted non-participant observations at each HASU at least four times, including two weekday visits, one evening visit during the week and 1 weekend visit, with additional visits as needed to confirm or add to our findings. Our observations were guided by clinical interventions in our quantitative analysis and our initial observations (see table 1), and we collected data on various aspects of HASU activity likely to influence care provision, including the work of the stroke team in the emergency department and on the wards. We were looking for patterns of usual care at different times of day and particularly how these differed (if at all) at night and at the weekends, for example, how clinical decisions were made, how meetings were conducted and so on.

Three researchers (GBB, JE and AB-L) conducted staff interviews. Participants were recruited through observation work and were provided with study information and assurance about the confidentiality of their participation. Sample size was determined in relation to qualitative research norms including for stroke^22–24^ and included a range of professional roles within all eight HASUs involved in the delivery of clinical interventions, such as medical, nursing, therapy and administrative or managerial staff (see table 2). Interviews were conducted following informed, written consent in private settings. Our semistructured topic guide included questions about temporal variations from the staff perspective, including typical daily activities, perspectives about patient care at different times of day and attitudes towards working patterns (see topic guide in the online supplemental file). Interviews lasted between 20 min and an hour. No field notes were taken, but each interview was discussed with the other team members. Interviews were audio-recorded and transcribed verbatim.

10.1136/bmjqs-2022-015620.supp1 Supplementary data

### Analysis

Four authors (GB, AIGR, AB-L and JE) initiated inductive coding of the interview and observation data while data collection was ongoing. From this we derived a group of codes relating to aspects of work at different times of day and days of the week in chime with the aims of our full project, following principles of thematic analysis.^16 25^ The coded data from each site were compiled in a spreadsheet to enable comparisons between sites and between professional groups. All authors discussed the analysis at regular project meetings for feedback, clinical input and in dialogue with ongoing quantitative analysis. For example, there were occasions where we felt we had witnessed temporal variation in observations that was not being measured in our quantitative dataset (our quantitative data are not reported in this paper). We developed our coding by triangulating information across the eight HASU sites and by referring to the documents which detailed the hyperacute stroke service standards. This helped us to understand whether practices we had witnessed were part of service specification (eg, timed targets for interventions) or locally derived (eg, rotas). The findings presented in this paper were derived from this primary analysis. We introduced the idea of temporal structuring following a literature search to help us understand and interpret the results.

## Results

**Figure 1:**
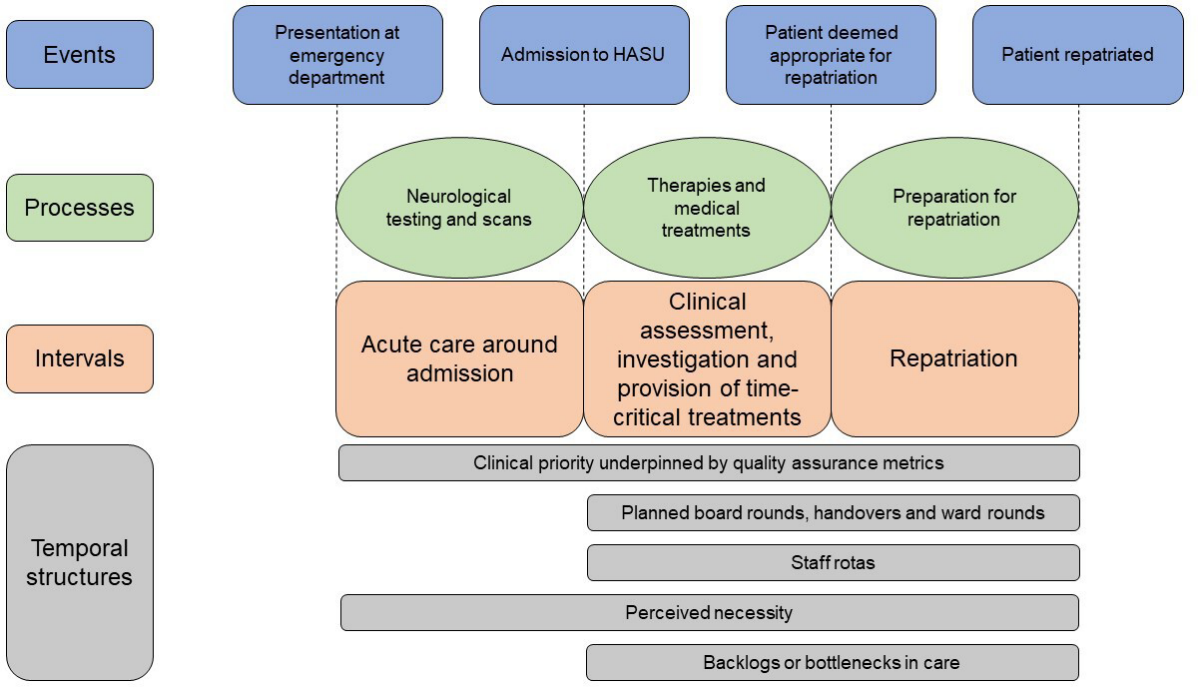
Patient-centred model of HASU pathway and temporal structures (after Scott *et al* 34 and adapted from Simister *et al* 16) of patients who had a stroke. HASU, hyperacute stroke unit.

Consistent with patient-focused studies, our observations and interviews characterised the HASU patient pathway in three main temporal intervals: (1) acute care, (2) assessment and stabilisation, and (3) repatriation.^26^ We derived five main factors that influence temporal structuring during these intervals (see figure 1). These include clinical priority underpinned by quality assurance metrics, planned meetings and visiting times, staff rotas, social norms about night and weekend activity, and after-effects or backlogs in care.

### Temporal imperatives: clinical priorities underpinned by quality assurance metrics create imperatives in the acute phase

The acute phase of the HASU pathway was critical to patient outcomes, and staff were strongly motivated by this and aware that they needed to work quickly. The HASU pathway was constructed to facilitate the timely delivery of stroke assessment, brain scanning, thrombolysis where appropriate, and screening for swallowing problems in keeping with both the London Stroke Standards and the National Clinical Guideline for Stroke (on which SSNAP is based). For example, the stroke team was alerted by a bleep when potential patients who had a stroke were on their way in an ambulance and could set up a prioritised CT scan:

They do prioritise a stroke patient, it’s why when we get a bleep to say we’ve got a patient coming we also have a CT to say, query thrombolysis, so they know that in a minute, that we might need to bring a patient scan… very rarely do we have to wait for CT because they prioritise all stroke patients. (H6, nurse)

All HASUs had a dedicated assessment team to meet the patient from the ambulance to immediately start their assessments. These clinical priorities were also underpinned by financial incentives. For example, the London Stroke Acute Commissioning and Tariff Guidance^21^ requires that HASUs meet temporal standards measured from ‘clock start’ (entrance to the hospital for patients outside hospital and time stroke symptoms spotted for inpatients), such as

- A ‘door to needle’ (thrombolysis) time of 45 min for eligible patients.
- Swallow screening within 4 hours.
- Patients scanned within 12 hours (although given the standard for thrombolysis, most patients will be scanned within 30 min to ascertain eligibility).

Participants confirmed that they were strongly motivated by this temporal imperative, particularly as it was something that they were judged on by other people:

On SSNAP, we don’t fail. So we hit the SSNAP targets, and really that’s what people measure you against (H4, speech and language therapist)

In contrast to the initial assessments and interventions, service standards in both the London Stroke Acute Commissioning and Tariff Guidance and the National Clinical Guideline for Stroke beyond the first 3 hours after onset are much broader. For example, patients are expected to see a consultant within 12 hours and a therapist within 72 hours post admission, with 45 min of therapeutic daily contact. These standards reflected social norms rather than research evidence; for example, the guidance was derived from a Cochrane review of occupational therapy interventions and a 2011 randomised controlled trial of neuropsychological therapy for patients who had a stroke;^27 28^ neither study specified a time interval within which interventions should be optimally initiated. The recommendations were also based on ‘working party consensus’, that is, the suggestions of the group developing the guideline. Interviews with therapists revealed that they were reluctant to change the targets because it could impact their personal lives, despite the fact that this was accepted for other professional groups:

So obviously people have family lives with children and obviously it’s their downtime at the weekends so I think, you know, thinking about how often you would have to work a weekend and how is that going to affect your quality of life. (H8, occupational therapist)

### Temporal rhythms: planned board rounds, handovers and ward rounds create work

Activities such as staff handovers, multidisciplinary team (MDT) meetings, the ward round and visiting hours patterned the day and determined the timing of clinical interventions. For a patient to receive evidence-based care, those moments of communication between all the staff members, the patients and their families were pivotal.

Every HASU observed had an MDT meeting (or ‘board round’) and a ward round every weekday and different arrangements on weekends. Individual disciplines had handovers between ending and starting shifts and team meetings to collate information before the board round to support decision making. Given the short intended patient stay, new admissions and potential discharges dominated board round conversations. In HASUs without a weekend board round, our interviews revealed that therapists sometimes overlooked patients due to a lack of prioritisation exercise (H2, H5 and H8).

The *ward* round was a daily event in HASUs starting in the morning and lasting 1–2 hours, where each patient was visited by a team of clinicians at the bedside. Some HASUs had a second ward round in the afternoon (H3, H7, and H8). Junior doctors, therapists and nurses described how the ward round acted as a stimulus for new work activities which filled the rest of the afternoon. Every ward also had restricted visiting hours for families, normally starting in the mid-afternoon. The arrival of families and friends while staff were trying to carry out these activities created an intense working phase. In one HASU, the nurse rotas were adjusted to account for this (H4), but consultants did not and were often still engaged in assessing new potential patients who had a stroke when patients and carers sought advice and discussion:

We’ve had feedback from the patients and families about access to the consultant. One of our problems is that you know, it’s very busy our HASU, so the consultant will do ward round in the morning, it takes them ’til 11 or 12 every day to finish the ward round […] and then they’ve got to do a follow-up ward round late afternoon to deal with all the new things that have happened during the day. So there’s a lot of pressure on them that takes them away from sitting down with the families and talking with them. (H2, consultant)

### Temporal peaks: staff rotas to meet demand

Rotas were constructed to meet peaks of activity on the ward. Nursing, medical and therapy rotas followed similar patterns on weekdays, with some small variations between HASUs (see table 3). We have not included weekend rotas as these were variable (eg, staff worked across two wards or were present only by telephone) but have reported on staffing details at night and the weekend in a previous study.^12^ In summary, the staff on the hyperacute stroke ward reduced both in number and seniority in the evenings and on weekends. We have discussed these patterns further.

**Table 3:** Weekday shifts by HASU and profession

HASU site identifier	Nursing	Medical	Therapy
H1	07.30–20.00	09.00–17.00	09.00–17.00
	19.30–08.00	17.00–21.00	
		21.00–09.00	
H2	08.00–20.30	09.00–17.00	09.00–18.00
	20.00–08.30	17.00–21.00	
		21.00–09.00	
H3	07.30–20.00	09.00–17.00	09.00–18.00
	19.30–08.00	17.00–21.00	
		21.00–09.00	
H4	07.30–15.30	09.00–17.00	09.00–17.00
	12.30–20.30	17.00–21.00	
	20.00–08.00	21.00–09.00	
H5	07.00–19.30	09.00–17.00	09.00–17.00
	19.00–07.30	17.00–21.00	
		21.00–09.00	
H6	07.30–20.00	09.00–17.00	09.00–17.00
	19.30–08.00	17.00–21.00	
		21.00–09.00	
H7	07.30–20.00	09.00–15.30	09.00–17.00
	19.30–08.00	14.00–20.00	
		20.00–09.00	
H8	08.00–20.30	09.00–17.00	09.00–17.00
	20.00–08.30	17.00–21.00	
		21.00–09.00	

This table is reproduced with permission from Simister *et al*.^16^

HASU, hyperacute stroke unit.

Bedside nursing was temporally structured to be constant, with most HASUs having two shifts; one HASU (H4) overlapped the early shift (07:30–15:30) and the late shift (12:30–20:30) so as to have the maximum staffing during the busy afternoon period. Weekend shifts were the same but often with a more junior nurse leading the ward or assessment team in the emergency department.

Therapists were mostly employed in standard 09:00–17:00 shifts on weekdays, but some HASUs experimented with extended shifts to 18:00. Two HASUs provided no therapy at all on weekends (H1 and H4). One HASU had a ‘voluntary’ therapy rota (H3). The other five HASUs provided a reduced therapy service compared with weekdays, for example, having either occupational therapy or physiotherapy but not both. Some had short shifts on weekends (seen as ‘skeleton’ rather than full cover). Participants expressed that therapists did not need to be on the ward at night; however, some felt there was potential value in extending therapy hours into the early evening. However, providing therapy at the weekend was normally achieved through overtime rather than increasing the number of employed therapists on the ward. Therefore, there were fears that a full 7-day model could make therapy staff ‘tired and miserable’ (H1, occupational therapist).

While consultants worked normative daytime hours, junior doctors covered day and night shifts. Consultants were physically present on the weekend and available by telephone at night. Our interviews showed broad acceptance of the lowered staffing and testing provision at night, and doctors in particular felt that they were not restricted in their management of a patient: ‘There is nothing I would want to have done that would be restricted based on time’ (H6, junior doctor). However, the correspondence between shifts and the times that medical staff would actually be on the HASU ward was less clear.

### Temporal heuristics: the perceived necessity rule

We discovered a dominant heuristic in how HASU staff perceived what was appropriate work for different times of day and days of the week: the *perceived necessity rule*. The perceived necessity rule indicated that on weekdays, staff should do anything that was required. On weekends and at night, they should do only what was necessary, defined by immediate risk to patients. This rule also meant that extra demands and processes were needed to achieve the same ends at night or on the weekend, compared with the week. For example, there was widespread acceptance that patients could have an MRI during the week to confirm their diagnosis but not over the weekend, despite the presence of radiology teams. Not all staff agreed with this perspective:

So I think there’s a bit of a culture change about, well, you know, it may not be a life-saving investigation but, you know, this allows the patient to be processed much more efficiently and quickly if it’s done now, rather than Monday, so it does seem a culture that you know, that can wait ‘til Monday because it’s not urgent or emergency. […] sometimes to get an opinion, a radiology … a consultant opinion, is quite difficult out of hours. […] It’s difficult enough in the week, actually, for us, but doing Saturday and Sunday, that just doesn’t happen. (H4, consultant)

During the daytime in the week, all patients suspected of having a stroke had some preferential access to scans and investigations by direct referral. At night and during the weekend, the decision whether or not to allow a patient who had a stroke to have an investigation was at the judgement of the consultant, however, and not a matter of hospital policy:

There’s probably one extra step on the phone call trying to organise the CT scan because you call the radiology registrar and explain that you want a scan rather than calling CT directly whereas during the day you tend to just order CT and they will get a radiologist to report your scan or they automatically get done during 9–5, you want a quicker result then you call them. But it’s more like a permission in a way because you know CT won't do it without a radiology registrar calling them and saying you can do a scan (H4, junior doctor)

There was widespread endorsement of the perceived necessity rule, so much so that certain participants lacked awareness of the differences, stating ‘We can get everything at any time’ (H3 consultant). However, junior doctors viewed this as problematic. Only one HASU was able to get MRI at night (H3), and five of the eight HASUs could access MRI at the weekend (H1, H2, H3, H4 and H7; some were restricted to a limited number of slots per day). Weekends were also characterised by the use of CT angiogram rather than carotid Doppler ultrasound studies to examine for carotid disease, driven at this time by the unavailability at weekends of ultrasound technicians to perform the Doppler studies. Carotid Doppler ultrasound is considered the first-line imaging in this context as it is non-invasive and carries no radiation risk in comparison to CT angiogram.

### Temporal after-effects: quality of care influenced by backlogs or bottlenecks

Quality of care provided to individual patients at any particular time was dependent on the after-effects of activities earlier in the day or on previous days. For example, backlogs in workload or bottlenecks in activities (eg, repatriating patients) were often caused by reduced or relatively junior staffing in previous days. Discharges that were planned during early in the day often extended into the evening, caused by waiting for hospital transport or for a family member to finish work. There were also late admissions to the ward which were prioritised, delaying consultant approval for discharge (see box 1).

Box 1 **Early evening observation notes relating to patient discharge**

In one early evening observation, we saw a patient who had hospital transport booked early in the morning, to arrive within a 90 minute window around 4pm. The nurses tried to co-ordinate with the patient’s care package, and as the transport ran later and later, the nurses became increasingly anxious about the possibility of him going home. Many phone calls were made to the transport provider and to the patient’s social worker. Despite the early morning booking, the patient could not be guaranteed a transport time because he was a low priority compared to patients with higher needs such as dialysis. The transport eventually arrived at 7pm but without the wheelchair that was ordered by the nurse. The social worker was still not contactable by telephone and the discharging nurse was concerned about the patient’s medication. They decided to give him the medication on the ward, and confirm that his wife and son were at home to receive him. In another evening observation, we saw a patient being transferred to a local stroke unit (SU) by ambulance as late as 9pm. There were concerns that the receiving SU would not have enough staff to accept the patient if the ambulance could not get there on time.

Backlogs were particularly notable on a Friday afternoon due to the pressue to create bed capacity for admissions over the weekend. It was also experienced on a Monday morning, when reduced services over the weekend had resulted in a backlog of work including new patients to be reviewed:

Mondays are horrendous […] the number of referrals varies quite wildly for us, but um, it can be up to sort of ten, some, some weeks. […] People don’t know the patients so well [on a Monday]. The information we get in Board Round is often quite confused. And so we, we, we kind of make a guess at referrals, we don’t always necessarily pick the appropriate one. (H3, speech and language therapist)

Therapists particularly noted that this influenced the type and quality of care they were able to provide. For example, rehabilitation therapy could be deprioritised in favour of meeting targets for first assessments.

There’s the sort of sudden stress of oh my goodness we’ve got fourteen new patients to see and when did they come in, when does their seventy two hours breach is you know a bit of a shock sometimes and then you end up pulling therapists off say the rehab ward to try and, so you don’t breach the seventy two hours. Then obviously the other patients get, you know they don’t have their forty five min that they’re meant to have and then there’s, we haven’t got enough wheelchairs for patients either, so sometimes we end up with patients sharing or just waiting on a waiting list which then is obviously not good for their rehab and therapy either. (H1, physiotherapist)

Some participants felt that this could delay patients’ recovery, particularly as some patients needed consistent, intense therapy every day to make progress.

### Illustrated example

**Figure 2:**
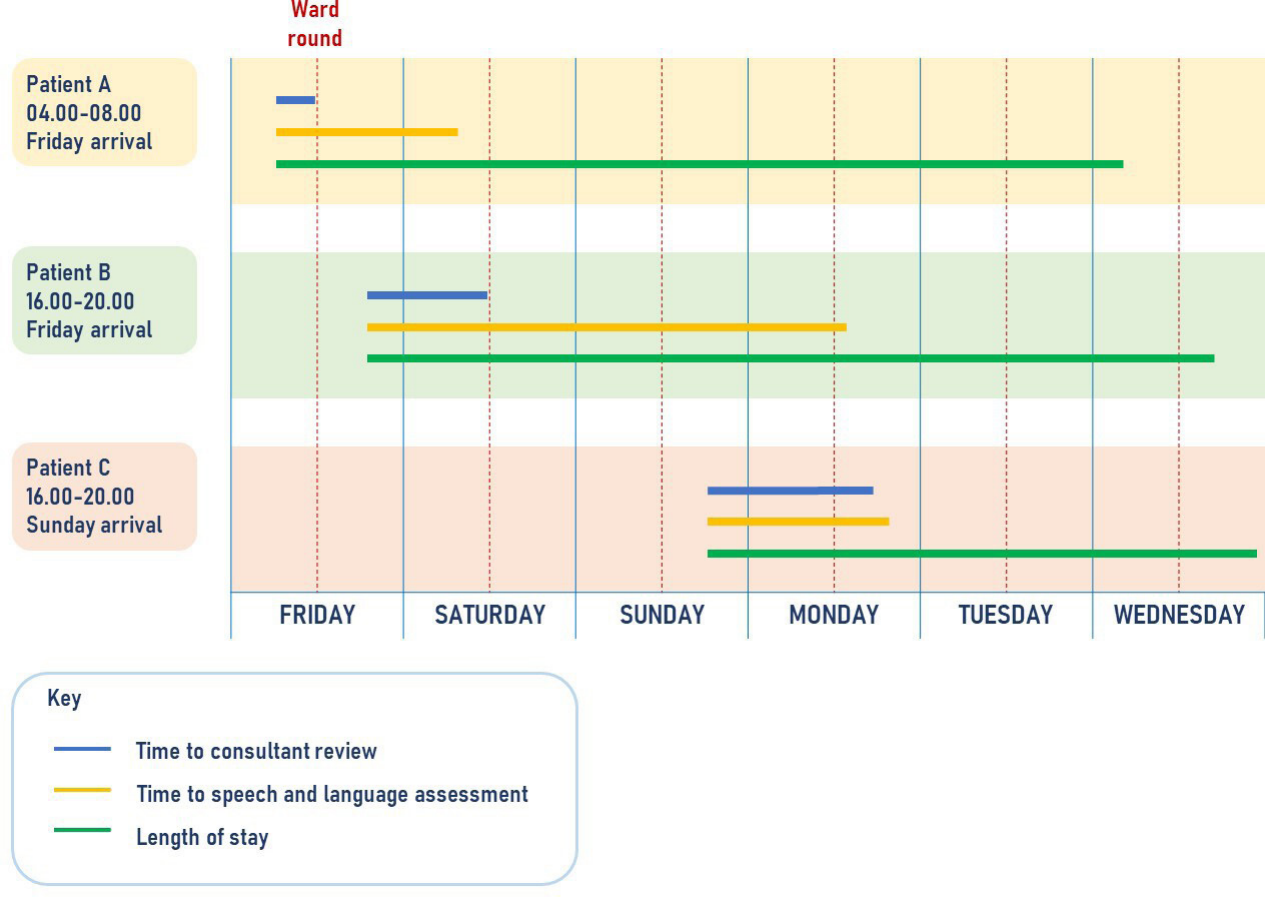
Examples of fictional patient trajectories and temporal variation in relation to time and day of admission based on median SSNAP data for time periods indicated (adapted from Simister *et al* 16). SSNAP, Sentinel Stroke National Audit Programme.

Figure 2 illustrates how the temporal structures reported previously may influence the timing of patient care. Patients A, B and C are fictional examples based on the *median time intervals for patients arriving in the time slots indicated from our SSNAP dataset*. Patients A and B both arrive on a weekday in hours. However, patient A sees a therapist after 24 hours and patient B sees a therapist after 60 hours because there are no therapists on the HASU over the weekend. Patient C arrives on a Sunday but sees a therapist relatively quickly when they return on Monday. According to London Stroke Acute Commissioning and Tariff Guidance and the National Clinical Guideline for Stroke, this is an acceptable result for all three patients. None of these measurements indicate quality of care; as the quotation from the speech and language therapist previously mentioned demonstrates, backlogs in patient care can challenge the amount of attention and information given about each patient. These after-effects are demonstrated in the time to discharge: patients A, B and C are all discharged on Wednesday.

## Discussion

### Summary of results

This study has described the temporal structures that influence timing of clinical interventions in hyperacute stroke wards. We generated five temporal structures that influence what-happens-when: (1) clinical priorities and quality assurance metrics motivate rapid activity for the initial life-prolonging assessments and interventions; (2) static features of ward organisation such as rotas and ward rounds have a strong impact on quality and consistency of care and determine the timing and quality of care for patients; (3) staff rotas which are subject to experimentation in some services to try to meet peaks in demand, although this was not always successful; (4) implicit social norms or heuristics about perceived necessity which affect staff motivation to make changes or improvements to consistency of care, particularly around weekend work; and (5) after-effects such as bottlenecks or backlogs affect quality of care, which are hard to measure effectively to drive service improvement.

### Comparison with previous literature

In consort with previous studies, our study suggests that ‘what-happens-when’ in stroke care and other health services is a combination of multiple human and system factors.^3 7^ We also concur that the weekend effect is a simplification, although there are substantial differences in staffing and organisation. Additionally, our findings highlight that backlogs arising from weekend staffing provision may cause weekday variation; quality of care may be lower on a Monday than on a Wednesday, for example. We have also highlighted daily variation, building on Bray *et al*, who examined quality of care in 4-hour blocks; workloads affected by ward rounds and rotas may shed light on these variations.^7^ Similarly, Shah *et al* noted variation in timely hip fracture surgery with day, evening and night effects, suggesting similar multifactorial after-effects to those found here.^29^ Other recent studies have looked at the impact of daily scheduled tasks such as breaks, staff meetings and clinical tasks on staff stress and burnout.^30^ Despite this, our previous paper found no significant variation in care quality across 42 time periods in any of the measures relating to brain scanning, stroke nursing care and thrombolysis.^13^

Our findings revealed contrasting stakeholder views about the acceptability and quality of care at weekends and at night. Bion and colleagues also found substantial variation between trusts in staff perception of quality of care in hospitals at the weekend.^31^

### Strengths and limitations

The strengths of this study are the rich nature of our dataset, including interviews, observations and our many discussions as a mixed methods research team. Some limitations should be noted: we did not gather any data in hyperacute stroke services operating within a different service model (whether centralised or non-centralised). Recent innovations such as thrombectomy will have introduced new temporal structures into the HASU pathway which were not captured in this study. We also omitted some relevant professions within the studied organisations (eg, pharmacy, emergency medical practitioners). This work was performed before the advent of COVID-19 and any changes to the functioning of hyperacute stroke services that may have arisen as a consequence of the NHS response to the pandemic. For example, there is evidence that services may now make more use of digital communication with an impact on ward routines.^32 33^ However, the impact of COVID-19 and the resultant understanding of the need for better resilience within the NHS in the face of further extreme stressor events emphasise the need for a better understanding of why care quality variation exists and of how to reduce it.

### Implications for policy and practice

Some of five factors we have described are organisational in nature; some are clinical; and others reflect social attitudes, which impact the possibility of improvement. For example, rotas are more amenable to change than long-held expectations about working patterns. External pressures, such as delays in discharge, may be outside the control of healthcare improvement efforts. Our study highlights the impact of organisational dynamics on quality of care: what happens on Sunday will affect the care patients receive on Tuesday. In the current resource-constrained environment, difficult decisions will be assessed against potential losses in care quality. Providing a reduced service in terms of diagnostic imaging or therapeutic input at the weekend may be a false economy if patients incur a longer stay in the hospital, for example.

Our findings suggest that the national stroke audit programme (SSNAP) model for reporting on service performance could be improved with more information on service performance variation. The clinical arm of SSNAP, which measures care delivered on every patient, is reported as a description of *average* service performance (eg, median time to delivery of thrombolysis or to first review by a physiotherapist) and on completion rates by the end of the admission (eg, proportion reviewed by an occupational therapist by 72 hours) rather than on the spread of performance over time or on the time to completion of assessments for some agreed majority proportion of eligible patients. Changes to the biennial organisational SSNAP audit could include measurement of cyclical variation in service structure, staffing and delivered care.

## Conclusions

Patients need temporally consistent quality of care supported by UK policy. Temporal consistency stems from the design of services, including staffing, targets and patient pathway design. To improve care, cultural values may need to change as well as financial investment; for example, improving therapy services at weekends will include the costs of additional posts and training and not just changes to rotas. Measuring temporal consistency of care quality should acknowledge how services are organised and not just rely on routinely collected or audit data.

## Data Availability

Data are available upon reasonable request. This paper draws on confidential interviews, which would not be appropriate for widespread sharing. However, the authors are able to share observation notes or excerpts of interviews upon reasonable request.
