# Supplementary material for: Temporal structures that determine consistency and quality of care: a case study in hyperacute stroke services
Source: BMJ Qual Saf. 2023 Jun 19;33(9):587–96. doi: 10.1136/bmjqs-2022-015620 (PMC11347214; doi:10.1136/bmjqs-2022-015620)
Supplement: Supplementary data [file bmjqs-2022-015620supp001.pdf]

UCL DEPARTMENT OF APPLIED HEALTH RESEARCH

HASU 24/7 study: Staff interview guide

1. Tell me about your background and role in the HASU.
- Position and length of time at HASU
- Previous experience with stroke care (and possibly other related fields, e.g. geriatrics)
- Shifts worked currently/previously worked in HASU

2. Tell me about a typical day working in the HASU.
- Explore each part of the day as described
- Managing tasks/demands (what helps)
- Challenges and where they come from

Listen/probe around:
- Description of procedures in line with clinical and organisational guidance for HASU
- Critical decisions: who, how, and when are they made
- Impact of how services are delivered on quality of care and patient/carer experiences
- Methods of working together/coordination of specialists
- Factors influencing HASU (e.g., hospital structure)
- Differences between in hours care and out of hours care

3. What is it like working 'out of hours', compared to 'in hours' at the HASU for you or your colleagues?
- Differences like**:** staff experience, quality of care, coordination, patient/carer experience
- Factors influencing differences
- Definitions of difference: OH weekend v. nightime
- Relevance of issue: OH/IH compared with other time-relevant differences (e.g., care delivered on different days of week) or factors perceived as more influential in shaping quality of care (e.g., staffing levels)

4. Tell me about how staff work together.
- Position and supervisory structure
- How disciplines are managed (e.g., separately or integrated; procedures for)
- Rota decisions: who makes and what influences in planning and implementing
- Rationale for arrangements
- impact of arrangements
- Differences between in hours care and out of hours care

5. In your experience, how does the HASU keep track of the quality of the care that it delivers?
- types of internal/external audit and assessment data used to track
- procedures for assessment
- staff roles and responsibilities
- extent to which IH/OH is assessed
- perceived impact of assessments
- important factors outside of HASU assessment (e.g., other hospitals' procedures)

Black GB, *et al. BMJ Qual Saf* 2024; 33:587–596. doi: 10.1136/bmjqs-2022-015620

6. What do you think is most important to delivering care successfully in the HASU?

-Follow key themes but listen for our key themes: time of care; staff coordination; quality monitoring; patient experiences; coordination of HASU within larger healthcare delivery systems and associated factors;

- factors/processes that influence ability to deliver

7. Is there anything else we have not discussed, that you would like to add?

Black GB, *et al. BMJ Qual Saf* 2024; 33:587–596. doi: 10.1136/bmjqs-2022-015620
